# Hypoxia Promotes Cartilage Regeneration in Cell-Seeded 3D-Printed Bioscaffolds Cultured with a Bespoke 3D Culture Device

**DOI:** 10.3390/ijms24076040

**Published:** 2023-03-23

**Authors:** Konstantinos Theodoridis, Eleni Aggelidou, Maria-Eleni Manthou, Aristeidis Kritis

**Affiliations:** 1 Department of Physiology and Pharmacology, School of Medicine, Faculty of Health Sciences, Aristotle University of Thessaloniki (A.U.Th), 54124 Thessaloniki, Greece; 2 CGMP Regenerative Medicine Facility, Department of Physiology and Pharmacology, School of Medicine, Faculty of Health Sciences, Aristotle University of Thessaloniki (A.U.Th), 54124 Thessaloniki, Greece; 3 Basic and Translational Research Unit (BTRU) of Special Unit for Biomedical Research and Education (BRESU), Faculty of Health Sciences, School of Medicine, Aristotle University of Thessaloniki (A.U.Th), 54124 Thessaloniki, Greece; 4 Laboratory of Histology, Embryology and Anthropology, School of Medicine, Faculty of Health Sciences, Aristotle University of Thessaloniki (A.U.Th), 54124 Thessaloniki, Greece

**Keywords:** cartilage, hypoxia, mesenchymal stem cells, TGF-β2, PCL 3D-printed scaffolds, 3D culture device and method

## Abstract

In this study, we investigated the effect of oxygen tension on the expansion of ADMSCs and on their differentiation toward their chondrocytic phenotype, regenerating a lab-based cartilaginous tissue with superior characteristics. Controversial results with reference to MSCs that were cultured under different hypoxic levels, mainly in 2D culturing settings combined with or without other biochemical stimulus factors, prompted our team to study the role of hypoxia on MSCs chondrogenic differentiation within an absolute 3D environment. Specifically, we used 3D-printed honeycomb-like PCL matrices seeded with ADMSCs in the presence or absence of TGF and cultured with a prototype 3D cell culture device, which was previously shown to favor nutrient/oxygen supply, cell adhesion, and infiltration within scaffolds. These conditions resulted in high-quality hyaline cartilage that was distributed uniformly within scaffolds. The presence of the TGF medium was necessary to successfully produce cartilaginous tissues with superior molecular and increased biomechanical properties. Despite hypoxia’s beneficial effect, it was overall not enough to fully differentiate ADMSCs or even promote cell expansion within 3D scaffolds alone.

## 1. Introduction

Articular cartilage, which is found mainly in diarthrodial joints, is a resilient tissue that under normal circumstances functions as a cushioning material. It is continuously exposed to mechanical forces during daily activities and it must withstand several times the body weight [1]. It has a very low self-regenerative capacity due to its avascular and non-innervated nature as a tissue, but unfortunately, it very commonly presents small lesions, given its key role as a shock absorber [2]. These lesions progressively lead to larger defects developing in the pathological modality of osteoarthritis (OA) [3]. OA is a serious chronic disease and is considered one of the leading causes of disability according to Centers for Disease Control and Prevention (CDC), and it is expected to increase, especially in the aging population [4,5].

The current treatments, such as microfracture and subchondral bone drilling, allogenic transplantations, autologous chondrocyte implantation, or even mesenchymal stem cells injections [6,7], can reduce pain and to some extent, can improve patients’ life quality; nevertheless, they are not capable of fully regenerating hyaline cartilage in articulating joints [8]. In most cases, mechanically inferior fibrocartilage and/or mixed tissue is produced, which carries the risk of failing in the long term. Furthermore, pertaining to chondrocyte transplantation, problems arise when trying to proliferate chondrocytes usually in monolayer cultures, because they tend to lose their chondrocytic phenotype and de-differentiate [9,10] The reported treatments are therefore useful, mainly for small cartilage lesions [11].

For cell-based therapies to reach their full potential, appropriate cell sources with adequate cell numbers, suitable scaffolds as 3D matrices, and ideal culture methods are required. Adipose mesenchymal stem cells can be obtained easily with minimal invasive procedures. MSCs have immunomodulatory and paracrine properties, thus reducing inflammation and helping the wound healing processes [12,13]. They also have the ability to yield large numbers ex vivo, without losing their stemness and multipotency at early passages [14,15]. Mesenchymal Stem Cells (MSCs) can further enhance cell–cell and matrix interactions within scaffolds and augment chondrogenic differentiation by mimicking the mesenchymal condensation observed during embryonic chondrogenesis [16] or when combined with other stimuli, such as growth factors (BMPs, IGF, FGF, or TGF) [17], oxygen tension [18], and mechanical stimulation [19].

Hyaline cartilage receives nutrients and oxygen from the synovial fluid by diffusion; therefore, chondrocytes, which are spread within the different zonal architecture of the tissue, are supplied with different oxygen pressures and are estimated to range from 10% O_2_ in the superficial zone to 1% O_2_ in the deeper zones [20,21]. In an attempt to mimic the physiological environment of the hyaline cartilage tissue, cells are cultured under hypoxic conditions, but the role of hypoxia on the proliferation and differentiation of MSCs is disputable, and most studies have been performed on monolayer cultures. Low oxygen tension was often shown to increase cell numbers and to better support cell colony formation, especially in monolayer cultures [22]. Some studies have reported that hypoxia induced chondrogenic differentiation, increased the collagen type II, sulfated glycosaminoglycan (GAGs) expression, and promoted the chondral extracellular matrix formation compared to normoxic conditions [22,23]. On the other hand, hypoxia has been reported either to impede in vitro terminal differentiation and hypertrophy of MSCs [24] or not to have a significant impact compared to normoxia [25], so the effect of oxygen tension remains vague for chondrogenesis and is still under study, specifically in 3D cultures with different scaffold architectures and materials.

Our study addresses a gap in our current knowledge on the role of hypoxia in MSC chondrogenic differentiation in a 3D culture environment. We used 3D-printed poly (epsilon-caprolactone) scaffolds seeded with adipose-derived mesenchymal stem cells (ADMSCs) to investigate the effect of oxygen tension on cell expansion and chondrogenic differentiation in the presence or absence of the growth factor TGF. For this purpose, we used a prototype 3D cell culture device that was previously shown to favor nutrient/oxygen supply, cell adhesion, infiltration, and proliferation within scaffolds, to gain the best performance on these factors. We aimed to investigate whether hypoxia alone can stimulate chondrogenesis and examine how its effect is altered by the presence or absence of TGF. We hope to add new information on the role of hypoxia in 3D cultures and to contribute to the design of more effective protocols for stem cell chondrogenic differentiation in hypoxic 3D cultures.

## 2. Results

### 2.1. Assessment of Live/Dead Cells

**Figure 1:**
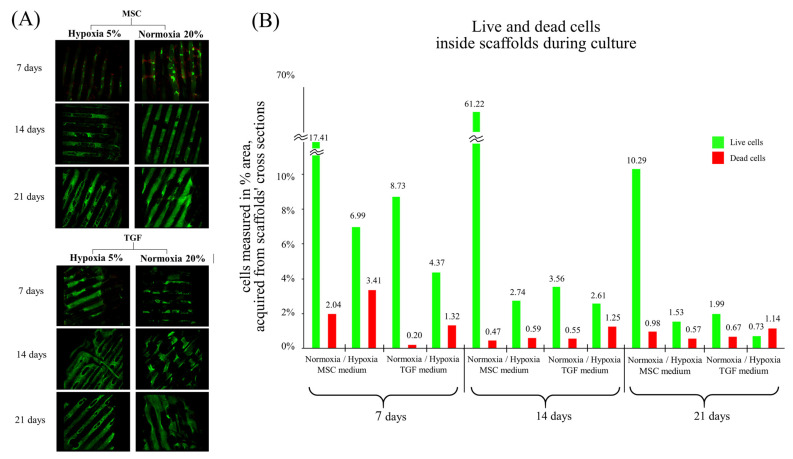
Live/dead fluorescent staining with calcein AM/ethidium homodimer. Live/dead images showed high levels of cell viability of the adipose-derived mesenchymal stem cells (ADMSCs) on polycaprolactone scaffolds cultured with the 3D-CD device (**A**). Live and dead cells inside scaffolds’ pore network were quantified by measuring the % area of cell coverage using Image J from cross-sectional images (supplementary material). The results are presented as a graph (**B**). All scaffold groups were evaluated on three critical time points: on the 7th, 14th, and the 21st day of cell differentiation. Images are merged for live and dead cells. Magnification ×4.

Cell viability was tested on the confocal microscope at three critical time points, the 7th day, the 14th day, and the 21st day of cell differentiation. The images were acquired from the top/bottom views of all scaffold variants (Figure 1) and from the cross sections (supplementary material).

On the top/bottom surfaces, the majority of cells remained viable at all the time points, with minor dead-cell batches and cell coverage increasing during culture period (Figure 1A). We compared the living cells/cell clusters between normoxia and hypoxia and between the MSC and TGF media on both time points of cell differentiation, i.e., the 14th and the 21st day, and found that the images were similar, indicating a matching cell development under all conditions.

Inside the scaffolds’ pore network, the living cells seemed to be much higher in number when cultured under normoxic conditions at all time points, with or without TGF. More dead cells were recognized under hypoxic conditions (Figure 1B). At every next timepoint studied, the live/dead cell ratio within scaffolds dropped in all cases. The highest viability and infiltration were recorded on the 14th day in normoxic MSC cultures.

Overall, the applied cell seeding method and culturing on our 3D-CD device resulted in a uniform cell coverage on all the scaffolds’ surfaces and a rather uniform cell migration inside all the pore networks.

### 2.2. Cell and Tissue Distribution, Gross Morphology and Macroscopic Characteristics of the Engineered Cartilage Tissue

**Figure 2:**
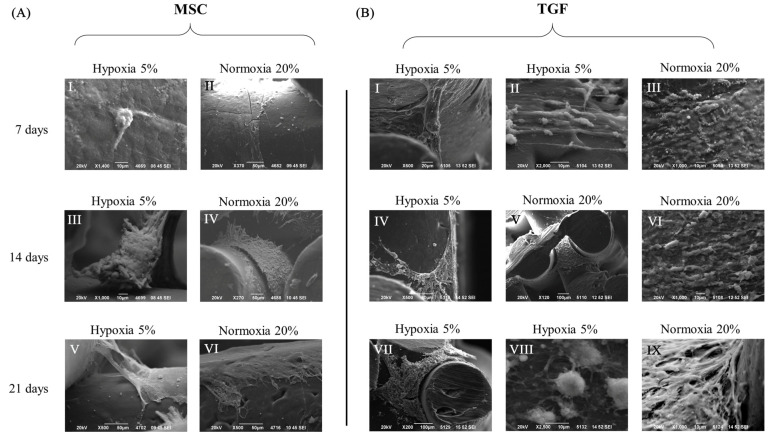
Scanning electron microscopy (SEM) of the adipose-derived mesenchymal stem cells (ADMSCs) on polycaprolactone scaffolds cultured with the 3D-CD device. All scaffold groups were evaluated on three critical time points: on the 7th, 14th, and the 21st day of cell differentiation. Group of scaffolds cultured under MSC medium with hypoxia are presented in (**AI**,**III**,**V**) and with normoxia in (**AII**,**IV**,**VI**). Group of scaffolds cultured under TGF medium with hypoxia are presented in (**BI**,**II**,**IV**,**VII**,**VIII**), and with normoxia in (**BIII**,**V**,**VI**,**IX**).

**Figure 3:**
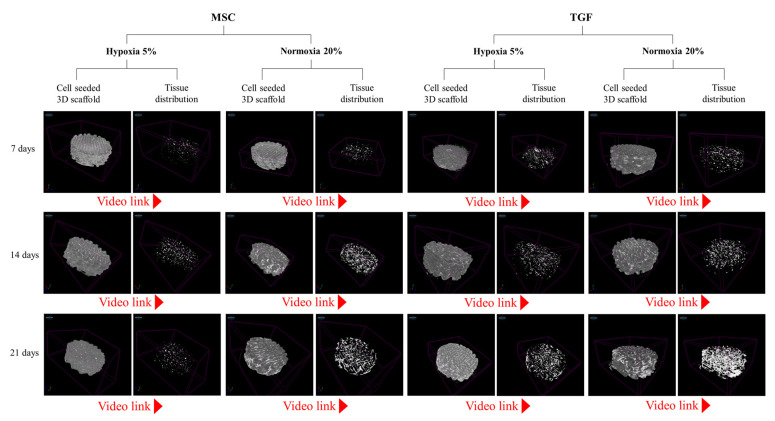
Micro-CT scans on polycaprolactone scaffolds cultured with the 3D-CD device. Images showing 3D volume renderings and video links (3D animation of the tissue ingrowth). Scanned specimens were created to represent tissue formation and distribution on three critical time points: on the 7th, 14th, and the 21st day of cell differentiation under MSC medium and/or TGF, in hypoxia and normoxia, respectively. Video animation can be visualized on the highlighted links https://www.mdpi.com/article/10.3390/ijms24076040/s1.

The external morphological characteristics of cells, of newly developed tissue, and of their association with scaffolds, were all assessed with micro-CT and SEM imaging at all time points under all experimental conditions (Figure 2 and Figure 3).

On the 7th day, SEM imaging showed that single cells or small cell clusters were attached and grew on the scaffolds’ fibers (Figure 2A,B). Most of the cells were fully attached on the fibers, while other group of cells suspended from above united with the group of cells proliferating immediately below, thus creating a columnar structure of cells that connected with the scaffold’s fibers (Figure 2(BI)). This was especially obvious within TGF scaffolds. Micro-CT scans additionally showed that the cells fully penetrated all scaffolds with a relatively uniform distribution.

On the 14th day of cell cultivation, SEM denoted a notable extracellular matrix (ECM) that extended inside the scaffolds (Figure 2A,B). Micro-CT images in the same timepoint showed that a more abundant ECM was documented in the MSC scaffolds compared to the TGF scaffolds, and in normoxia compared to hypoxia (Figure 3).

On the 21st day of the culture, both SEM and micro-CT indicated that normoxia was still more favorable than hypoxia for tissue formation. Interestingly, TGF scaffolds at this time performed better than MSC scaffolds, appearing with denser and more abundant ECM (Figure 2 and Figure 3). A closer look of cells in SEM showed that they were round and larger, measuring approximately 10 μm in diameter (Figure 2(BVIII)).

### 2.3. Histochemical Evaluation of the Engineered Cartilage Tissue

We studied the sections of all scaffolds on the 7th, 14th, and 21st day of culture under all experimental variants.

**Figure 4:**
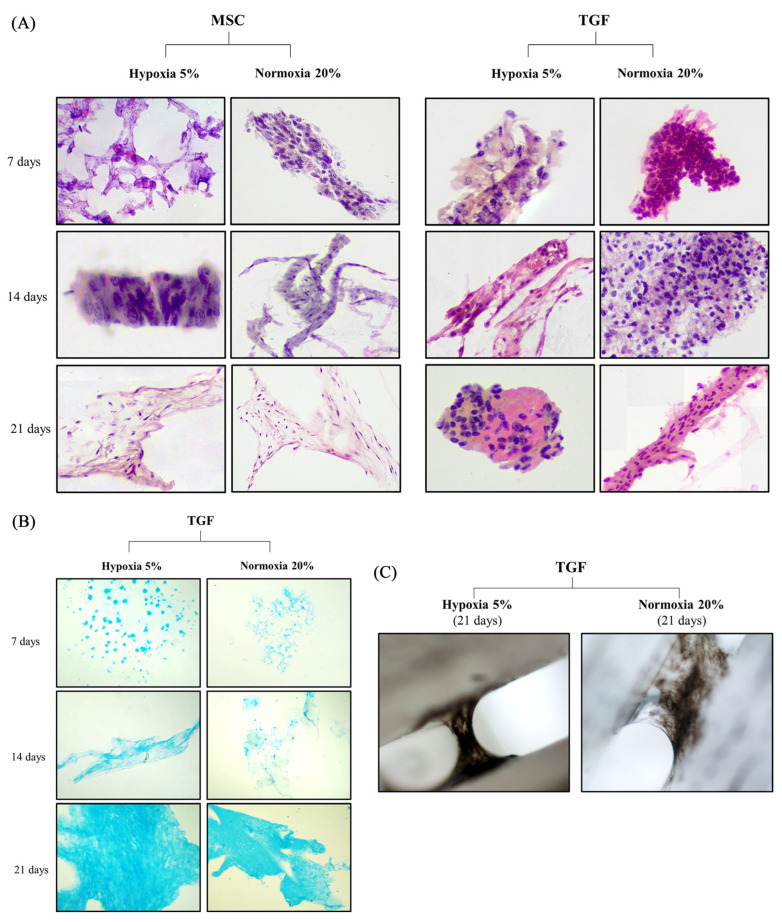
Histochemical evaluation of adipose-derived mesenchymal stem cells (ADMSCs) seeded on polycaprolactone scaffolds and cultured with the 3D-CD device under MSC or TGF medium (TGF-β2) in normoxic and hypoxic conditions. Hematoxylin and eosin staining confirmed that ADMSCs were more mature after 26 days of culture in TGF medium than those cultured in MSC medium (**A**). Proteoglycan synthesis was confirmed by Alcian blue staining, indicating that GAG content in ADMSCs was greater in scaffolds cultured in TGF medium and in hypoxic conditions (**B**). Scaffolds stained with Indian ink after 21 days cultured in TGF medium indicate tissue formation of rich collagen content in both normoxic and hypoxic conditions (**C**). Hematoxylin and eosin, magnification ×40; Alcian blue, magnification ×40; Indian ink, magnification ×10.

On the 7th day, the cells appeared relatively sparse in small groups, with little cytoplasm and small, round nuclei. More abundant cells were found on TGF-N scaffolds, which appeared very close to each other, forming small three-dimensional groups (Figure 4A).

On the 14th day, sheets of round and more mature cells were observed, with more extracellular matrices present. In addition, in hypoxic scaffolds treated with TGF, collagen fibrillation within ECM formation was more common. Furthermore, Alcian blue coloring negatively charged ECM, indicating the production of ECM with more proteoglycan content compared to scaffolds on the 7th day (Figure 4B).

On the 21st day, round cells with more extracellular matrices present, especially in TGF-H scaffolds, were observed in H&E images. Closely situated, spindle-shaped cells resembling the perichondrium were found in all TGF scaffolds, as well as a higher proteoglycan content, as indicated by Alcian blue (Figure 4B). TGF scaffolds were also positively stained with Indian ink, indicating the formation of rich collagen content (Figure 4C).

### 2.4. Chondrogenic Evaluation of the Engineered Cartilage Tissue

The tissue that was developed from ADMSCs seeded on PCL scaffolds was further evaluated with RT-qPCR and three major markers (SOX 9, collagen type II, and collagen type I) to compare the chondrogenic differentiation potential of the chondrogenic medium (TGF) and of normoxic or hypoxic conditions. 

**Figure 5:**
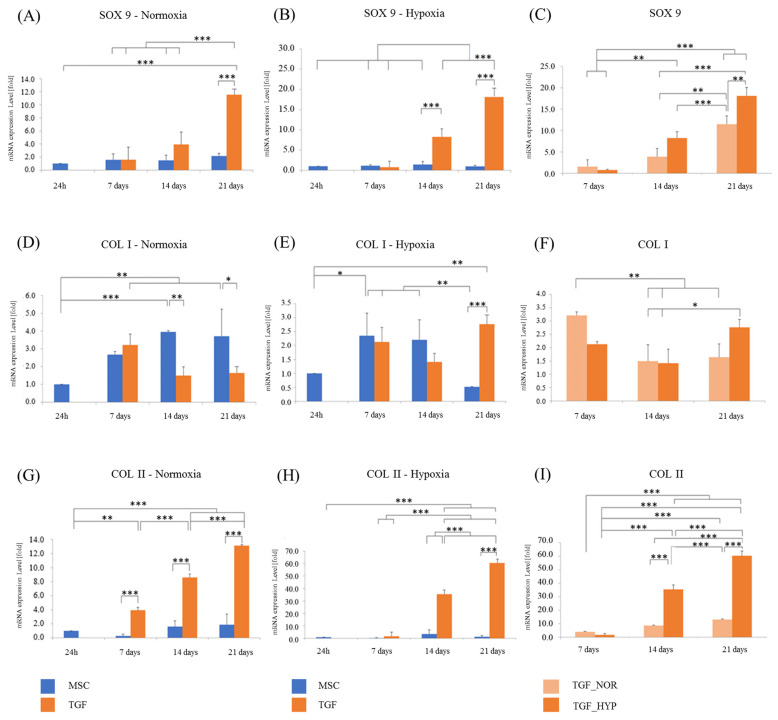
Real-time PCR analysis of the expression of chondrogenic differentiation markers, SOX9, COL I, and COL II in ADMSCs/scaffold constructs on three critical time points, 7th, 14th, and 21st day of cell differentiation: MSC and TGF media and hypoxia and normoxia. (**A**,**D**,**G**) Expression of the chondrogenic markers SOX9, COL I, and COL II respectively, after 1, 7, 14- and 21-days expansion with MSC and TGF culture media under normoxic conditions. (**B**,**E**,**H**) Expression of the chondrogenic markers SOX9, COL I, and COL II respectively, after 1, 7, 14- and 21-days expansion with MSC and TGF culture media under hypoxic conditions. (**C**,**F**,**I**) Expression of the chondrogenic markers SOX9, COL I, and COL II respectively, after 7, 14- and 21-days expansion with TGF culture media under normoxic and hypoxic conditions. SDHA2 was used as housekeeping gene control. Values are means of n = 3 (±SD); * *p* < 0.01, ** *p* < 0.001, and *** *p* < 0.0001.

SOX-9, a master transcription factor in chondrogenesis that indicates early chondrogenesis, was upregulated significantly only in scaffolds treated with TGF under both normoxic and hypoxic conditions. Cells cultured for 21 days in normoxia exhibited a 11.5 ± 1.92-fold increase in their expression of SOX-9 (Figure 5A). Interestingly, the same increase was achieved much earlier in hypoxia, around the 14th day of culture (8.28 ± 1.47-fold), and by the 21st day, the expression had almost doubled (18.12 ± 1.97-fold) (Figure 5B). On the other hand, the MSC medium failed to support early chondrogenesis, as demonstrated by the very low levels of SOX-9 at all time points.

Collagen type II, which is the most abundant collagen type in mature articular cartilage, was only significantly expressed by cells in TGF scaffolds. Under normoxic conditions, an increase was noticed already on the 7th day (3.94 ± 0.39-fold), reaching 13.13 ± 0.49-fold on the 21st day (Figure 5G). Cells in the TGF-H scaffolds exhibited their first significant increase in collagen type II expression on the 14th day, reaching a 35.42 ± 3.4-fold increase on day 14, and a 60.43 ± 3.6-fold increase on day 21 (Figure 5H). Both hypoxic and normoxic conditions resulted in a significant increase in the Coll II expression in TGF media; nevertheless, hypoxia led to a much better result, which was almost five times higher an expression than that of normoxia (Figure 5I). Once again, the MSC medium did not seem to support chondrogenesis, and the expression of Coll II was very low at all time points.

On the other hand, collagen type I, which is more abundant in fibrous tissue and fibrocartilage, was generally expressed at very low levels. In the MSC media, the highest expression of collagen type I was observed under normoxic conditions on day 21 (3.7 ± 1.52-fold) (Figure 5D–F), while the lowest expression was detected in hypoxia on the 21st day (0.52 ± 0.01-fold).

### 2.5. Biomechanical Analysis of the Engineered Cartilage Tissue

**Figure 6:**
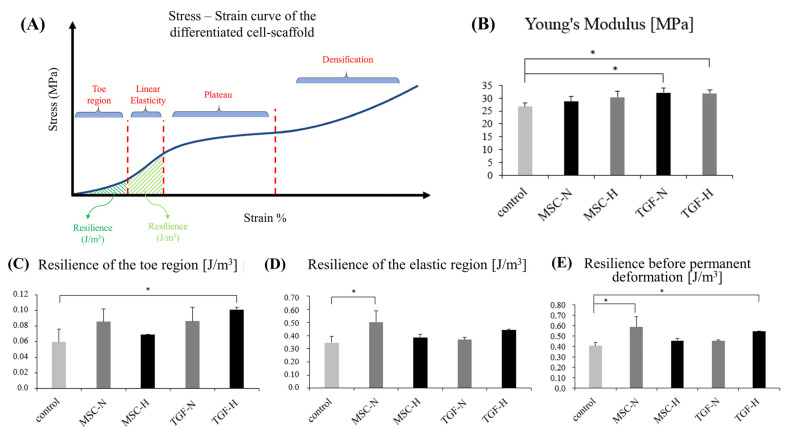
Unconfined compression tests were performed under a uniaxial compression testing system (Instron 3344, Instron^®^) with 1 kN load cell in accordance with ASTM F451 guidelines. 10-mm-diameter scaffolds were tested at the end of the culture period (21 days of cell differentiation) to investigate the mechanical impact of the newly formed tissue in all experimental variants. Stress–strain curve characteristics (**A**) and compressive Young’s modulus in MPa (**B**). Biological responses of the regenerated tissue as a material, in terms of absorbed energy (J/m^3^) in the toe region (**C**) and the elastic region (**D**). Total energy absorbed as a resilience measurement before permanent deformation (**E**). Values are means of n = 3 (± SD); * *p* < 0.01.

Numerical data collected from the stress–strain curves are shown in Table 1. Null scaffolds displayed a compressive Young’s modulus of 26.66 ± 1.54 MPa. By day 21, the stiffer scaffolds were the scaffolds cultured in the TGF medium, under both the normoxic (20.5% increase) and hypoxic conditions (19.35% increase) (Figure 6B). A similar increase was also found in the MSC groups of the scaffolds, but with no statistical significance.

The energy absorbed in the toe region of the null scaffolds was 0.059 ± 0.016 J/m^3^. The number was increased by ~71% in the TGF-H scaffolds and by ~46% in the TGF-N scaffolds (Figure 6C). The energy absorbed in the elastic region was statistically increased the most on MSC-N scaffolds (Figure 6D). The total resilience before deformation was increased by 44.5% in the MSC-N scaffolds and by 34% in the TGF-H scaffolds (Figure 6E).

## 3. Discussion

In this study, the effect of oxygen tension on the expansion of ADMSCs and on their differentiation, with or without TGF medium, toward a chondrocyte phenotype was examined. Hyaline cartilage tissue was developed in 3D scaffolds from mesenchymal cells, applying a prototype method that was established in our own previous experiments, which was shown to favor cell colonization and infiltration with minimal cell loss. Newly formed tissues developed and were distributed uniformly within the scaffold. Hypoxia seemed to favor cell differentiation, especially when combined with TGF, but it did not seem to promote cell expansion. Overall, in all the scaffolds and under all the conditions at all time points, cell adhesion, infiltration, proliferation, and viability were kept at high levels.

### 3.1. Hypoxia and Chondrogenesis

There is a discrepancy in the literature concerning whether hypoxic conditions are beneficial to chondrogenesis or to the maintenance of the chondrocytic phenotype [22,23,24,25,26,27,28,29]. Normally, chondrocytes in articular cartilage grow and mature in a hypoxic environment, so it is only natural to speculate that hypoxia probably acts favorably. We aimed to mimic the natural hypoxic environment of hyaline cartilage tissue in vitro and to study the contribution of hypoxia in terms of committing ADMSCs to the chondrogenic lineage.

Our data show that neither normoxia nor hypoxia alone are enough to induce an up-regulation of chondrogenic markers during prolonged culture. More specifically, hypoxia alone could not induce the expression of SOX9 and collagen type II, and after 21 days in culture, it significantly prevented the osteogenic/fibroblastic marker collagen type I from increasing. Hence, hypoxia reduces the production of a fibroblastic ECM, which is quite important when developing articular cartilage for clinical applications. When combined with TGF, hypoxia proved to be more favorable than normoxia. More specifically, hypoxia led to a five times higher increase in collagen type II at 21 days. The level of expression of SOX9 in hypoxia at 14 days was reached much later under normoxic conditions at 21 days. Overall, hypoxia exerted a positive effect on cells, but only when combined with a TGF medium did it lead to a chondrocytic phenotype. In this case, differentiation is performed sooner than in normoxia, with higher levels of collagen type II.

In a study using PLLA scaffolds and mesenchymal cells, hypoxia was tested with a TGF medium and was compared to normoxia [30]. At 21 days, SOX9 and collagen type II were expressed at higher levels in hypoxia, which is similar to the results of our study. Hypoxia was set at 2%, which led to a 2.5-fold higher level of collagen type II in hypoxia compared to normoxia. In our study, the respective ratio was five times higher, which may be attributed to our lower hypoxic conditions, which were set to 5%, or to the different culturing method used with the described device, or both.

In another study, where hypoxia was set at 3%, the mesenchymal cells were allowed to differentiate in the TGF medium, and they were compared to normoxia [31]. Collagen type II expression started increasing on the 3rd day of hypoxic culture, whereas in normoxia, the first detection of collagen II occurred on the 14th day of culture, which is in accordance with our study.

Hypoxic conditions are shown to act beneficially not only in 3D scaffolds but also in other kinds of 3D cultures. The expression of collagen type II increased at all time points (7, 14, and 21 days) in hypoxia compared to normoxia, which was observed in a study performed on chondrospheroids [13].

On the other hand, there is a study where hypoxia was not shown to favor the differentiation of mesenchymal cells when compared to normoxia. In this study, with collagen sponges and umbilical cord MSCs [32], there was no significant difference between hypoxic and normoxic cultures in the expression of SOX9 at all time points (7, 14, and 21 days), either with or without TGF. In our study, SOX-9 was upregulated significantly in scaffolds treated with TGF under both oxic conditions, but it was especially favored by hypoxia. Interestingly, the same increase in SOX-9 levels was achieved much later in normoxia than in hypoxia. In the study of Leduc et al., it is reported that the TGF medium caused an increase in the collagen II expression at 7 days, which was not further enhanced by any type of oxic condition. In our study, it is obvious that hypoxia exerted a positive role in collagen II expression after the 7th day. More specifically, the levels of collagen II were five times higher under hypoxia compared to normoxia.

### 3.2. Hypoxia and Cell Expansion

In our study, hypoxia overall did not act beneficially on the total cell number compared to normoxia, which is in agreement with other studies [31,33,34].

However, not all studies have similar results. The viability of human adipose stem cell spheroids was recorded in a study by Zubillaga et al. under normoxic and hypoxic (<5%) conditions at three time points (7, 14, and 21 days) and with a chondrogenic medium [13]. More dead cells were observed under normoxia at all time points, which is in contrast to our study. In the study by Zubillaga et al., the spheroids were subsequently added to 3D porous chitosan and chitin nanocrystal scaffolds, and adhesion was also examined. A larger number of cells egressed from the spheroids, colonizing a greater extent of the biomaterial surface under hypoxia compared to normoxia. In our study, it was hypoxia that caused more dead cells within the scaffolds in contrast to normoxia, but the conditions of the two studies are obviously very different.

Interestingly, the cell response under low oxygen tension was shown to depend on the type of scaffold used, such as PCL, PCL-hyaluronic acid (HA), and PCL-Bioglass (BG) [35]. Low oxygen tension had a positive effect on cell proliferation, mainly in BG and HA scaffolds. Mesenchymal stem cells seemed to display certain material-dependent responses to hypoxia, but the overall proliferation was slower in hypoxic conditions than in normoxia up to day 17, except for the hyaluronic acid samples, where proliferation was comparable in both oxic conditions [35]. Our study showed that the hypoxic conditions had a negative effect on cell expansion at all the time points and until the end of the culture (21 days), mainly inside the PCL scaffolds. In a study with hydrogels, the effect of hypoxia on cell behavior compared to normoxia was investigated on both softer and stiffer scaffolds [36]. It was shown that hypoxia significantly increased the percentage of cells in colonies on both soft and stiff scaffold surfaces and promoted colony formation. Additionally, hypoxia led to an increase in the spread area of mesenchymal stem cells on soft surfaces.

Various hypoxic levels (0.1, 1, 2, 3, 4, or 5%) were tested in a study, and the proliferation of human mesenchymal stem cells was compared in monolayer cultures [37]. On the first day after cell seeding, cell proliferation was significantly higher in cells cultured at 1% of O_2_, and it was kept rather high until the end of culture. Overall, at all time points and under all oxic conditions, cell proliferation was comparable. Cells cultured under normoxia rapidly proliferated in the first three days, but on the fourth day, the proliferation rate decreased. By day 14, normoxia exhibited the lowest cell proliferation compared to 1, 2, 3, and 4% of O_2_. In our study, the results are different, with normoxia having a positive effect on proliferation beyond the third day, but the oxic conditions in 2D and 3D cultures should ideally not be compared.

Hypoxia seems to have a beneficial effect on cell expansion, mostly in monolayer cultures [22,38,39,40,41,42]. This might be explained by the fact that the low oxygen tension applied remains constant at every part of the culture, exerting the same continuous effect on all cells. When scaffolds are involved in a study, it is reasonable to expect that the oxygen pressure outside the scaffold or on the scaffold’s surface might not be the same as the pressure within and can vary significantly, especially when comparing different kinds of scaffolds and 3D culture methods.

### 3.3. Biomechanical Properties

A successful engineered cartilage tissue should have three major characteristics. The newly formed tissue must have a chondrogenic phenotype, fill a great part of the scaffold, and display ideal mechanical properties. Normally, articular cartilage withstands various loadings on a daily basis. There are joints carrying up to 1.5 times the bodyweight, such as the shoulders, while ankles can carry 2.5 times, and hip and knee joints can reach up to 3.5 times the bodyweight [1].

In this study, we applied a newly developed method to produce superior engineered cartilage tissue bearing the previously described three major characteristics. We performed destructive compression testing on the cartilage because it was shown to be well suited for the determination of the properties of the collagen network. The compression applied was unconfined, which increased the pressure within the tissue and resulted in the lateral stretching of collagen fibers, eventually leading to their breakdown [43]. All scaffolds produced at the end of culture, under all conditions, exhibited an increase in the compressive modulus. The highest increase was documented in the scaffolds cultured in a TGF medium in both oxic conditions. Both hypoxic and normoxic scaffolds displayed ~20% upregulation of the compressive modulus, reaching approximately 32 MPa, a value much higher than normal. Studies have shown that the normal values generally range between 2.6 and 18.6 MPa [1,44,45].

The resulting increased stiffness is reasonably attributed to pronounced ECM production rich in collagen type II, to proteoglycan synthesis, and to the extended filling of the scaffold with newly formed tissue. The proteoglycans are reported to be mainly responsible for the biomechanical characteristics of compression [46,47,48], but the collagen network also plays an important role in the stiffness of the final construct. When compression is applied, there is fluid movement inside the tissue, which causes pressure differences and results in the swelling of the tissue. The swelling is restrained by the collagen network, which results in high levels of stiffness [49].

## 4. Materials and Methods

### 4.1. Cell Isolation 

Cells were isolated from human resected abdominal flap tissue during abdominoplasty procedure (Papageorgiou Hospital, Hospital Review Board-approved protocols 263-7/12/2016) and with the patient’s consent. Adipose tissue was minced into pieces and enzymatically digested in a mixed solution of 4 mg/mL collagenase/dispase (Roche, Basel, Switzerland) for 5 h at 37 °C. Cells were isolated and expanded in an MEM medium (Invitrogen, Waltham, MA, USA) mixed with 15% *v/v* FBS (EU-tested, Invitrogen) 2 mM Glutamine, 0.1 mM L-ascorbic acid phosphate (Sigma-Aldrich, Taufkirchen, Germany), 100 U/mL penicillin, and 100 mg/mL streptomycin (all from Sigma-Aldrich). In this study, we refer to this home-made medium as MSC. Isolated cells were expanded until passage 3, and characterized as Mesenchymal Stem Cells by a Guava^®^ easyCyte 8 HT flow cytometer (Merck-Millipore, Darmstadt, Germany), similarly to our previous work [50]. Cells expressed CD90 and CD73, whereas CD45 (BioLegend, San Diego, CA, USA) was used as a negative marker.

### 4.2. Scaffolds and Culturing Device 

**Figure 7:**
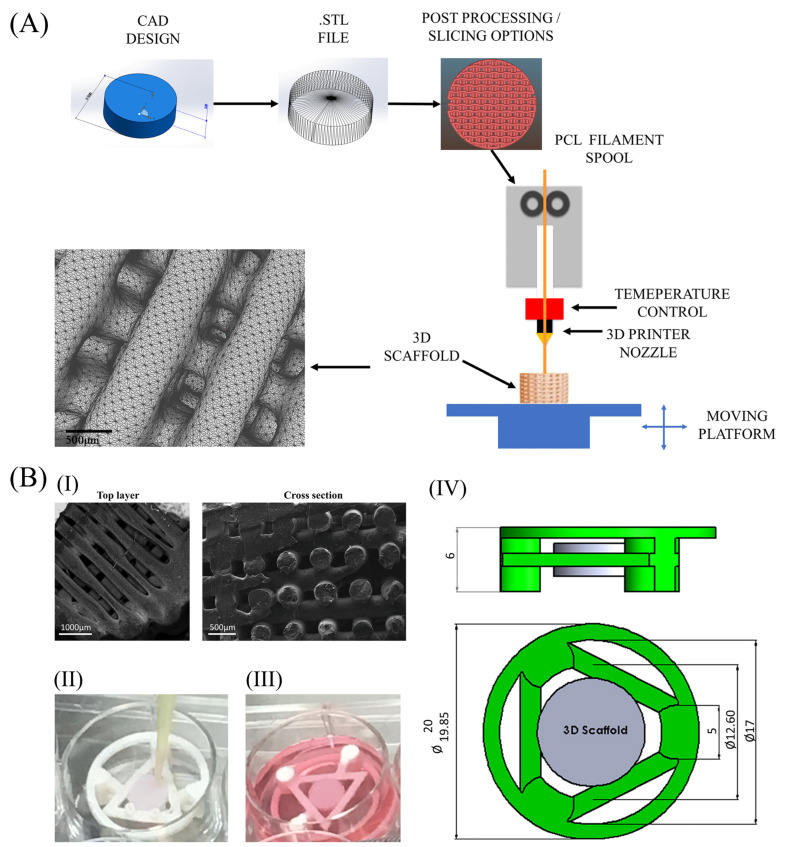
Scaffold fabrication process (**A**). Scaffold pattern and porous structure (**BI**), Three-dimensional cell culturing method and device(**BII**,**III**,**IV**). Scaffolds fixed on the 3D-CD device and cell seeding (**BII**), scaffolds supplemented with culture media (**BIII**), and 3D-CD device dimensions (**BIV**).

Poly (epsilon-caprolactone) or PCL scaffolds (PCL, 3D4MAKERS, Haarlem, The Netherlands, MW = 50 kDa) were fabricated by a desktop 3D printing machine (PRUSA i3, Newark, NJ, USA), as illustrated in the diagram (Figure 7A). Scaffolds were designed with a honeycomb-like pattern, incorporated by squares and hexagonal pore shapes, with an average pore size of 425 μm and porosity of 83.4 (Figure 7(BI)). This architecture was chosen amongst several tested in previous, own studies, where cell adhesion, proliferation and chondrogenic differentiation were compared and evaluated [51,52]. Scaffolds were cylindrical in shape, with 10 mm diameter, 3 mm height, and they comprised of 10 layers which were each 300 μm thick. The extrusion temperature was set to 145 °C and the speed to 60 mm/s. A total of 100 scaffolds were fabricated for this experiment. After production, all scaffolds were immersed in 4 M NaOH to enhance their hydrophilicity and to be cleaned. Scaffolds were exposed to UV irradiation for 30 min for extra sterilization. Prior to cell seeding, all scaffolds were further incubated with MSC medium for 24 h in the incubator.

Additionally, we fabricated a specially designed device, named 3D-CD (3D culturing device), which can hold scaffolds away from the surrounding walls, suspending them within the well. The hover-scaffolds promote better cell adhesion, proliferation, and internal cell distribution and ensure negligible cell loss during seeding, according to previous results [52]. The device was designed to fit in 6-well plates (Figure 7(BIV)) and was manufactured with the same benchtop 3D-printing machine that was used to fabricate the scaffolds. Poly (lactic acid) (PLA), (3D4MAKERS, Netherlands) was used as material for this device. 100 such devices were produced and were immediately sterilized by immersion in ethanol for 30 min and by exposure to UV irradiation for another 30 min. Subsequently, all scaffolds were fixed to the devices, as shown in Figure 7(BIV).

### 4.3. Cell Culturing Conditions 

Devices holding scaffolds were transferred to a 6-well plate for cell seeding. Cells measuring 1.5 × 10^5^ were seeded to each scaffold (Figure 7(BII)). More specifically, half of the cells were suspended in 10 μL MSC medium on the top side of each scaffold and were incubated at 37 °C for 60 min. The remaining cells were seeded on the bottom side of each scaffold and incubation continued for another 60 min. 1.5 mL of MSC medium was then added to each well (Figure 7(BIII)). After seeding, cells proliferated for a 7-day period within the incubator, under normoxic conditions (20% O_2_ and 5% CO_2_), while nutrients were changed every 2 days. Scaffolds remained on the device for 21 more days after proliferation period in two different culturing media to enable cells to differentiate into cartilage tissue. Half of the scaffolds were left to differentiate in a home-made chondrogenic medium, which we named TGF. It is based on α-MEM, with the addition of 15% FBS, 0.1 mM L-ascorbic acid phosphate, 2 mM glutamine, ITS, 100 nM dexamethasone, 10 ng/mL transforming growth factor-β_2_, 100 U/mL penicillin, and 100 mg/mL streptomycin (all from Sigma-Aldrich). The remaining scaffolds were cultured in MSC medium, as previously described. Two different culturing conditions were tested on the scaffolds, normoxic (20% O_2_ and 5% CO_2_) and hypoxic (5% O_2_ and 5% CO_2_), designated as -N and -H, respectively. Four different groups of scaffolds were therefore created, MSC-N, MSC-H, TGF-N, and TGF-H, which were all examined at 3 separate time points after the end of proliferation period, 7, 14, and 21 days.

### 4.4. Cell viability and Confocal Microscopy 

Viability/Cytotoxicity Assay kit (Biotium, CA, USA) was used for the documentation of both live and dead cells, according to the manufacturer’s instructions. Briefly, cell-seeded scaffolds were double stained with calcein AM and ethidium homodimer, they were covered to keep the light away, and they were placed at room temperature (RT) for at least 30 min before they were visualized with a confocal upright fluorescent microscope (Nikon D-Eclipse 80i C1, Tokyo, Japan). EZ-C1 3.20 software was used to capture Z-stack images. Additionally, cross-sectional images of the scaffolds were evaluated with image J for cell viability and migration within the scaffold’s pore network. Briefly, images of sections were uploaded to image J and converted to 8-bit images [53,54]. A common scale was set for all images, and a clear distinction between the objects of interest (stained cells) and the material was made by thresholding the pixels of each image in the same way. Finally, the mean value of cell area coverage was measured separately for both live and dead cells [55]. All scaffold groups were evaluated at three critical time points: the 7th, 14th, and 21st days of cell differentiation.

### 4.5. Scanning Electron Microscopy

Cells and regenerated tissue were observed morphologically by means of SEM. The scaffolds were fixed with 3% *v*/*v* glutaraldehyde (grade I), rinsed, and post-fixed with osmium tetroxide, rinsed again, and then they were dehydrated in increasing concentrations (30–100% *v*/*v*) of ethanol in water. The scaffolds were then dried, sputter coated with carbon, and observed under SEM microscope (JEOL JSM-6390 LV, Tokyo, Japan) at an accelerating voltage of 20 kV. All types of scaffolds from all three time points were studied.

### 4.6. Micro-Computed Tomography

SkyScan 1172 micro-tomograph (Bruker, Kontich, Belgium) was used to create a projection of cells and regenerated tissue growing within scaffolds. This scanner uses a tungsten X-ray source and is equipped with an 11 MP CCD camera (4000 × 2672 pixels). All specimens were scanned at a voltage of 59 kV and 167 μA. Camera was without filter; it was fully rotating 360° at the highest resolution. SkyScan’s NRecon software (NRecon, Bruker, Kontich, Belgium) was used to reconstruct sliced projected images (6 μm/slice) into cross-section images by implementation of a modified Feldkamp’s back-projection algorithm. All scans were loaded into the CTVox software (CTVox, Bruker, Kontich, Belgium) to render the proportion of the area occupied by the cells and the regenerated tissue throughout the scaffold’s architecture. Animated videos and images were created to visually represent the tissue formation inside scaffolds. Scaffolds under all described experimental conditions and at all three time points were studied with micro-CT.

### 4.7. Histochemical Evaluation

Cell-seeded scaffolds from the three chosen time points (7, 14, and 21 days after proliferation period) were fixed in 10% *v*/*v* neutral buffered formalin overnight at RT. After fixation, scaffolds were dehydrated in graded ethanol series, immersed in a toluole bath for 5 min, and then embedded in paraffin at 55 °C for 60 min. After blocking, paraffin cubes were cut transversely into 6 μm thick sections using a Leica RM 2235 rotary microtome. Selected sections, from equal distances throughout the whole scaffold, were heated at 60 °C for 30 min, deparaffinized, hydrated, and then stained with Hematoxylin and Eosin (H&E). Sections were dehydrated and covered. Similar protocol was also used for Alcian Blue staining (solution: 1% *w*/*v* pH = 2.5, adjusted with 3% glacial acetic acid of quality level 300, Sigma-Aldrich). After 21 days in culture, semiquantitative evaluation of collagen development was performed by staining the regenerated ECM in scaffolds with Indian ink. Ink has the appropriate chemical and physical properties to stain collagen within tissues [51,56]. PBS was mixed with black Indian ink (2:1) for 2 min and then gently applied to the scaffolds with a 22-gauge needle. Stained scaffolds remained in RT for 5 min to equilibrate and were then immersed in PBS 3 times. Scaffolds were then placed on slides and examined with a Nikon H550s microscope.

### 4.8. Quantitative Real-Time Reverse Transcription Polymerase Chain Reaction

RT-qPCR was used to compare the chondrogenic differentiation potential of cell-seeded scaffolds cultured in MSC and TGF media under normoxic or hypoxic conditions. For baseline gene expression, lysates were obtained from expanded cells at day 1 after culture in MSC medium. Three time points were selected for evaluating chondrogenic differentiation: 7, 14, and 21 days. One scaffold yields sufficient RNA for one biological replicate. Three biological replicates for each experimental condition per time point was used in this study. More specifically, total RNA was extracted from the constructs using the Nucleo-ZOL (Macherey Nagel, Düren, Germany), according to the manufacturer’s instructions. The RNA purity and concentration were measured using a NanoDrop spectrophotometer (Epock BioTek, BioTek Instruments, Inc. Headquarters, Santa Clara, CA, USA). The obtained RNA samples were then reverse transcribed using a SuperScript First-Strand Synthesis Kit (Takara Bio USA, Inc.), according to the manufacturer’s instructions. Reactions were performed using the SYBR-Select PCR Master Mix (Applied Biosystems, Foster City, CA, USA) in a StepOnePlus thermal cycler (Applied Biosystems). All reactions started with two initial incubation steps at 50 °C for 2 min for uracil-N-glycosylase activation and at 95 °C for 2 min for activation of the AmpliTaq DNA polymerase. 40 cycles of PCR are followed, comprising denaturation for 15 s at 95 °C and annealing/extension for 1 min at 60 °C. Primers were designed using the PRIMER BLAST for the following chondrogenic genes: SOX9 F: AGGAAGTCGGTGAAGAACGG, R: CGCCTTGAAGATGGCGTTG, COLI F: CGAGGCTCTGAAGGTCCC R: CAGGAGCACCATTGGCAC, and COLII F: AGGAGACTGGGTGGGATTCT R: GGTGAGCCCAGCTTACTCAG. The results were adjusted for amplification efficiency (LinRegPCR) and normalized against one housekeeping gene (succinate dehydrogenase complex, subunit A, flavoprotein-SDH-A). F:GCATGCCAGGGAAGACTACA R:GCCAACGTC CACATAGGACA found to remain stable during differentiation processes of cells.

### 4.9. Biomechanical Properties 

Unconfined compression tests were performed on the 10 mm diameter scaffolds at the end of the culture period in all scaffold variants to investigate the mechanical impact of the newly formed tissue. For each culture variant, three scaffolds were tested under a uniaxial compression testing system (Instron 3344, Instron^®^) with 1 kN load cell in accordance with ASTM F451 guidelines. Prior to testing, specimens were immersed in PBS for 2 h to allow equilibration, and then they were compressed in the Z-direction at a crosshead speed of 1 mm/min, in RT (~25°C). Bluehill 2 Universal software (Instron^®^) was used for the measurements up to a specimen’s strain level of approximately 75%. Young’s modulus (MPa) was calculated using a secant slope within the 10% range of the linear regime of the stress–strain curves (Figure 6A). While the curve shifts to the plateau region (Figure 6A), compressive strength (σ_y_) is calculated at the intersection point with a modulus slope at an offset of 1% strain. Additionally, an effort was made to investigate different biological responses of the regenerated tissue in terms of absorbed energy (J/m^3^). Two areas of biological interest on the stress–strain curve were measured: the toe region and the linear region. The two areas were also measured as an overall area, which does not exhibit permanent deformation of the material (Figure 6A). The strain area underneath the curve in these regimes was integrated, and the energy absorbed at each stage was calculated.

### 4.10. Statistical Analysis

Statistical analysis was carried out using GraphPad Prism software (version 5.00; GraphPad Software, San Diego, CA, USA). Data are presented as mean ± standard deviation (SD). Comparisons among groups were performed using one-way ANOVA, followed by post hoc Tukey’s multiple comparison test (mechanical properties). Gene expression was analyzed under the experimental factors of oxygen level (normoxia and hypoxia), culture condition (presence or absence of TGF), and time (baseline and days 7, 14, and 21) using two-way ANOVA with post hoc Tukey multiple comparison. Each combination of the experimental conditions was performed in triplicate. Probability values (*p*) inferior to 0.01 (∗), 0.001 (∗∗), or 0.0001 (∗∗∗) were considered to be statistically significant and marked in the figures accordingly.

## 5. Conclusions

The prototype culture method applied in this study acted beneficially on cell colonization and infiltration, exhibiting minimal cell loss. In all scaffolds, under all conditions and at all time points, cell adhesion, infiltration, proliferation, and cell viability were kept at high levels. Hyaline cartilage of a high quality, as indicated by molecular markers and histological examination, was developed in 3D scaffolds from mesenchymal cells under hypoxia. The newly formed tissue was distributed uniformly within the scaffold; nevertheless, hypoxia did not seem to promote cell expansion. The possibility of applying hypoxia a few days later, aiming to ensure a larger initial cell population before the differentiating effect of low oxygen pressure begins, should be considered.

Depending on the pore network, cell coverage, and cell infiltration, deeper parts of the scaffold will have lower than the applied oxygen tension, since the interconnected architecture will create extra hypoxic conditions than those of the incubator settings. As a result, the reproducibility effect of such experiments is rather limited. Furthermore, it is not possible to achieve an oxygen tension gradient similar to the in vivo physiological conditions, which is a limitation of this study. Despite its beneficial effect, hypoxia alone was not enough to form fully differentiated hyaline cartilage. The presence of TGF medium was necessary to successfully produce cartilaginous tissue with superior biomechanical properties. The molecular characteristics of the produced tissue were very satisfying; however, we could also try to additionally involve other possibly favorable factors, such as the application of mechanical pressure during culture, which will simulate natural conditions.

## Figures and Tables

**Table 1:** Mechanical properties.

Scaffold Groups	Young’s Modulus (MPa)	Yield Strength (MPa)	Resilience of the Toe Region J/m^3^	Resilience of the Elastic Region J/m^3^	Resilience before Permanent Deformation J/m^3^
Control	26.66 (±1.54)	4.47 (±0.21)	0.059 (±0.016)	0.347 (±0.047)	0.406 (±0.030)
MSC-N	28.83 (±1.81)	5.41 (±0.62)	0.085 (±0.017)	0.501 (±0.086)	0.587 (±0.100)
MSC-H	30.38 (±2.39)	4.83 (±0.06)	0.069 (±0.000)	0.386 (±0.023)	0.455 (±0.023)
TGF-N	32.13 (±1.74)	4.98 (±0.24)	0.086 (±0.018)	0.368 (±0.018)	0.454 (±0.009)
TGF-H	31.82 (±1.26)	5.39 (±0.05)	0.101 (±0.003)	0.443 (±0.005)	0.544 (±0.002)

## Data Availability

All data associated with this work are in the figures or supplementary material.

## Supplementary Materials

The following supporting information can be downloaded at: https://www.mdpi.com/article/10.3390/ijms24076040/s1.
