# Supplementary figures and images for: Hypoxia Promotes Cartilage Regeneration in Cell-Seeded 3D-Printed Bioscaffolds Cultured with a Bespoke 3D Culture Device
Source: Int J Mol Sci. 2023 Mar 23;24(7):6040. doi: 10.3390/ijms24076040 (PMC10094683; doi:10.3390/ijms24076040)

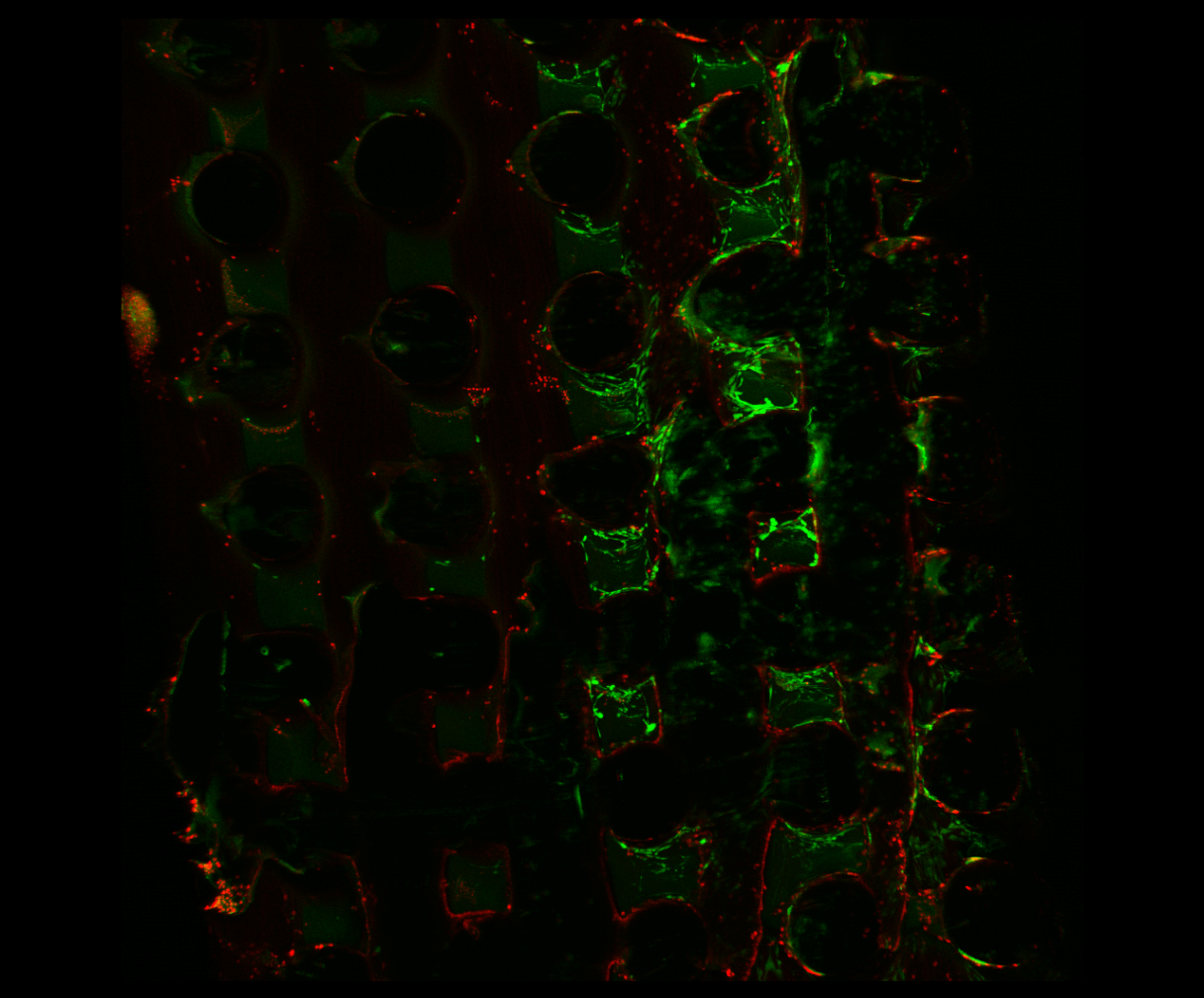

Supplement: Supplementary file 1 [file ijms-24-06040-s001.zip › MSC-H_14Days_Live-Dead.tif]

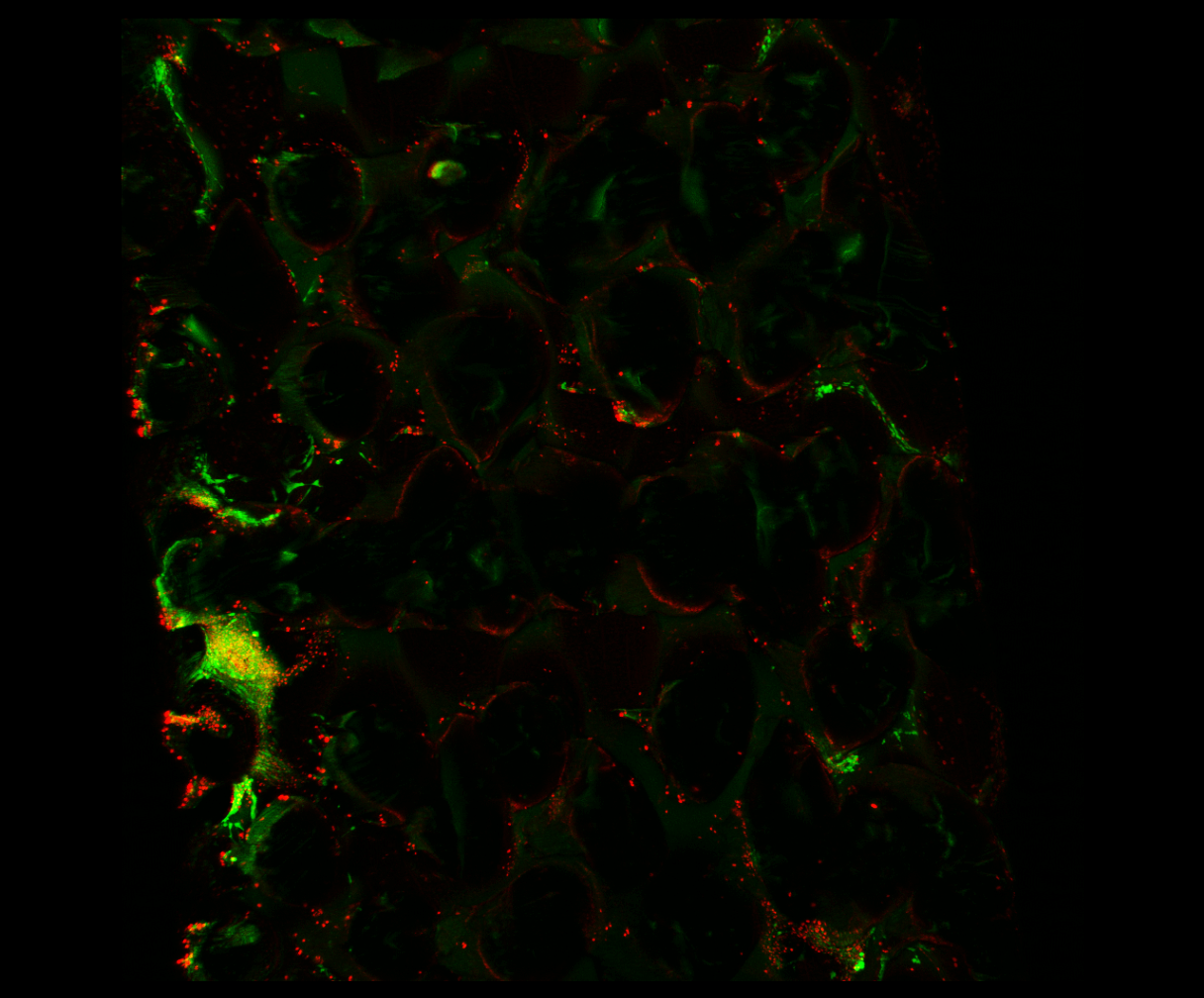

Supplement: Supplementary file 1 [file ijms-24-06040-s001.zip › MSC-H_21Days_Live-Dead.tif]

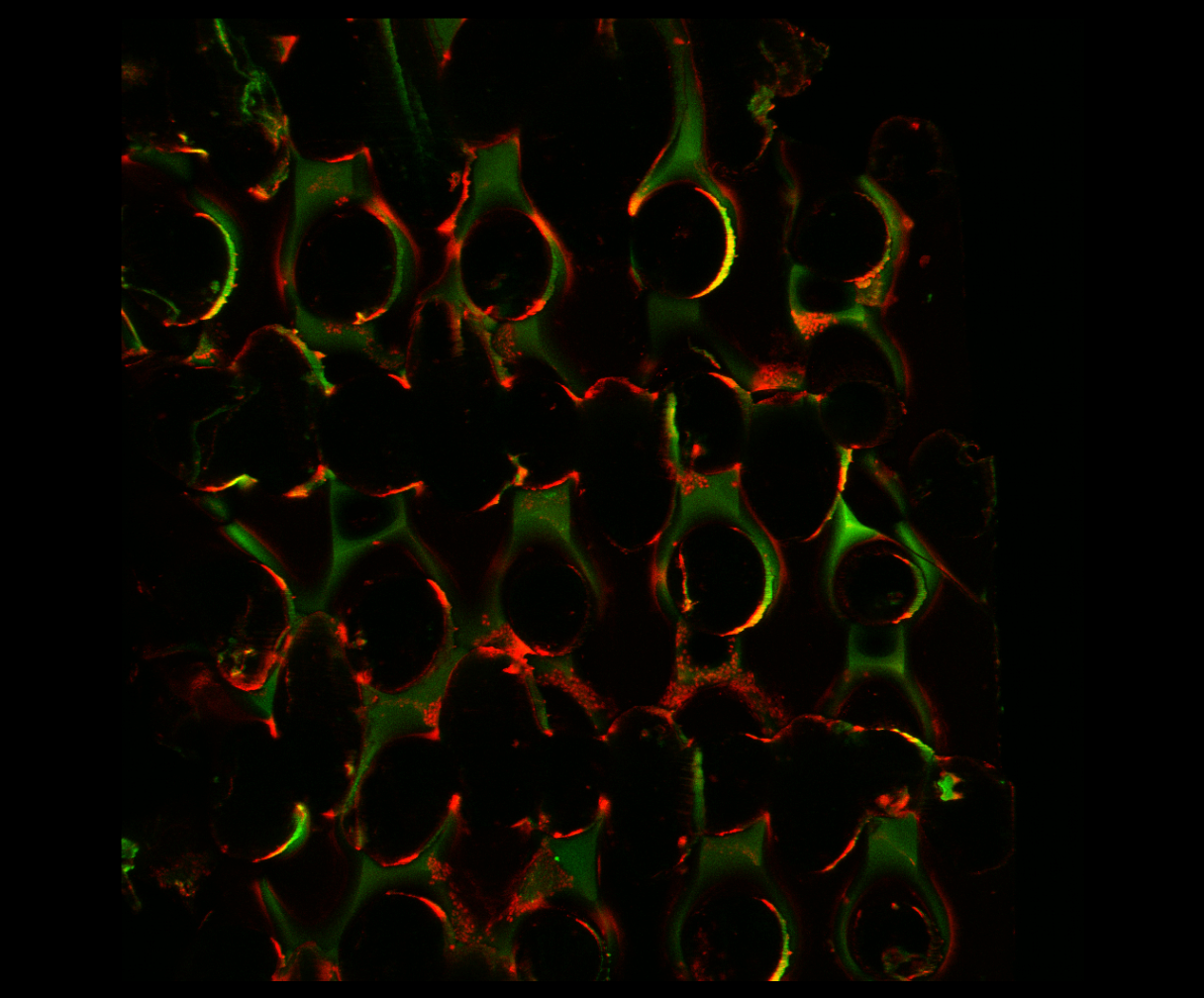

Supplement: Supplementary file 1 [file ijms-24-06040-s001.zip › MSC-H_7Days_Live-Dead.tif]

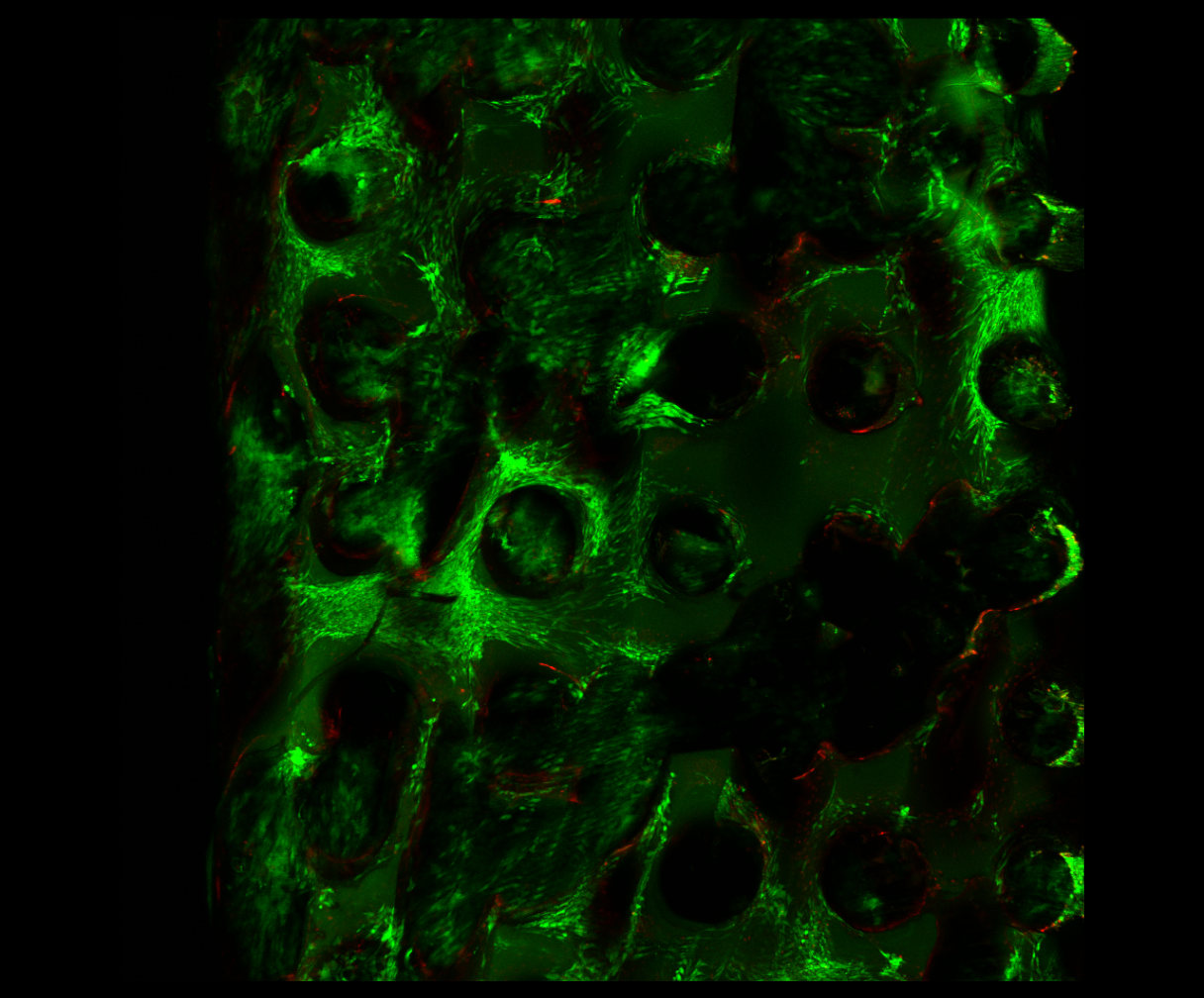

Supplement: Supplementary file 1 [file ijms-24-06040-s001.zip › MSC-N_14Days_Live-Dead.tif]

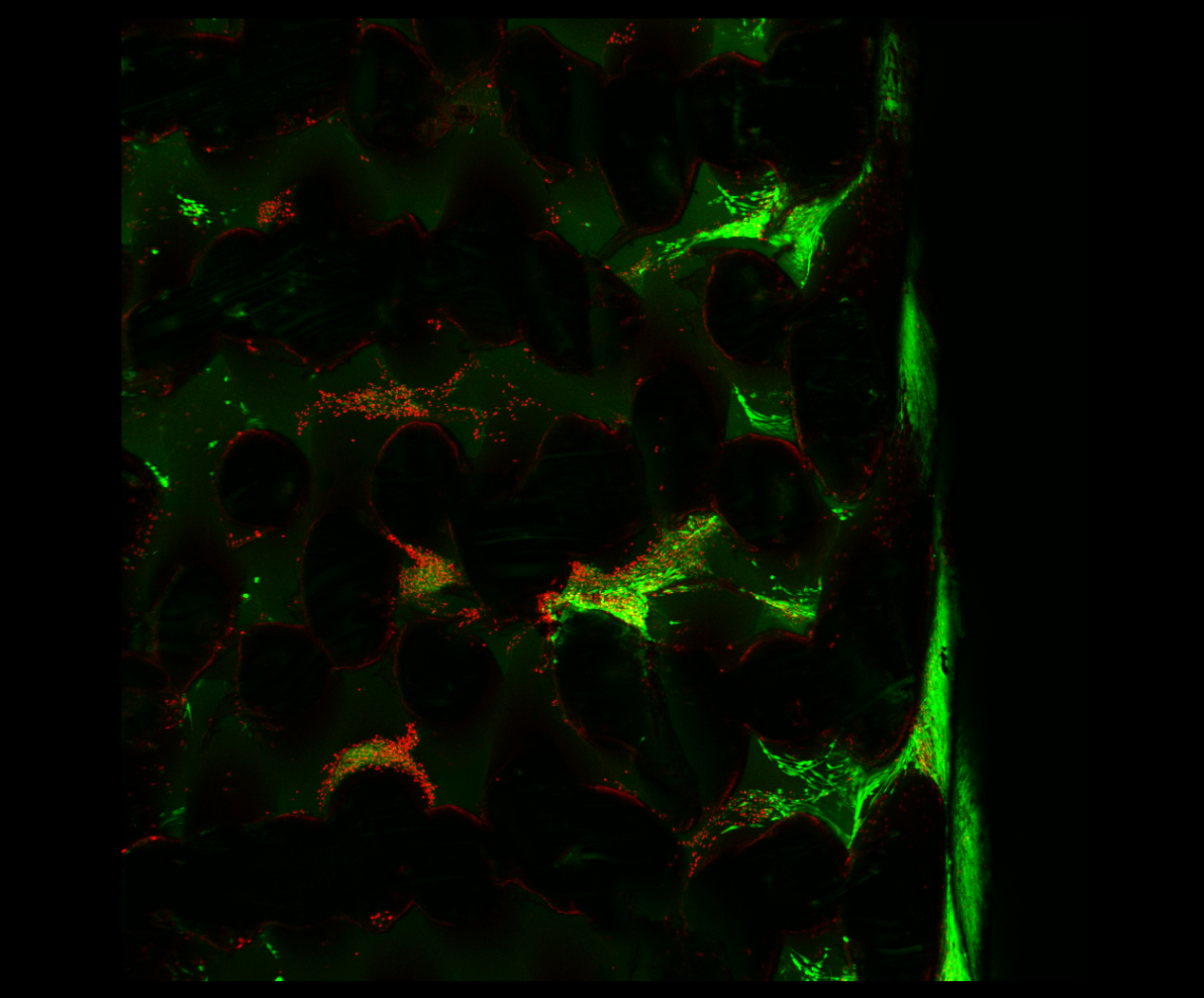

Supplement: Supplementary file 1 [file ijms-24-06040-s001.zip › MSC-N_21Days_Live-Dead.tif]

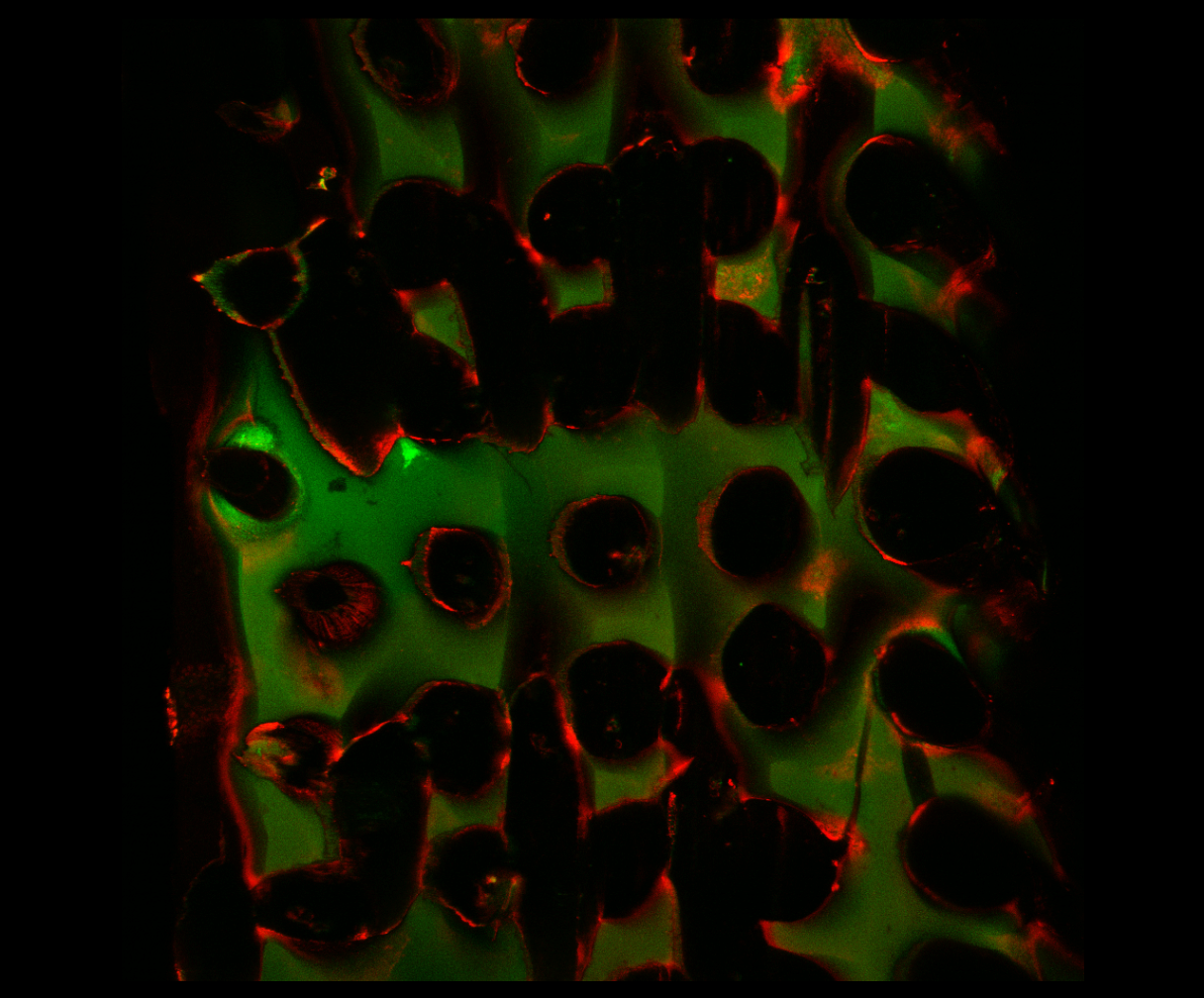

Supplement: Supplementary file 1 [file ijms-24-06040-s001.zip › MSC-N_7Days_live-Dead.tif]

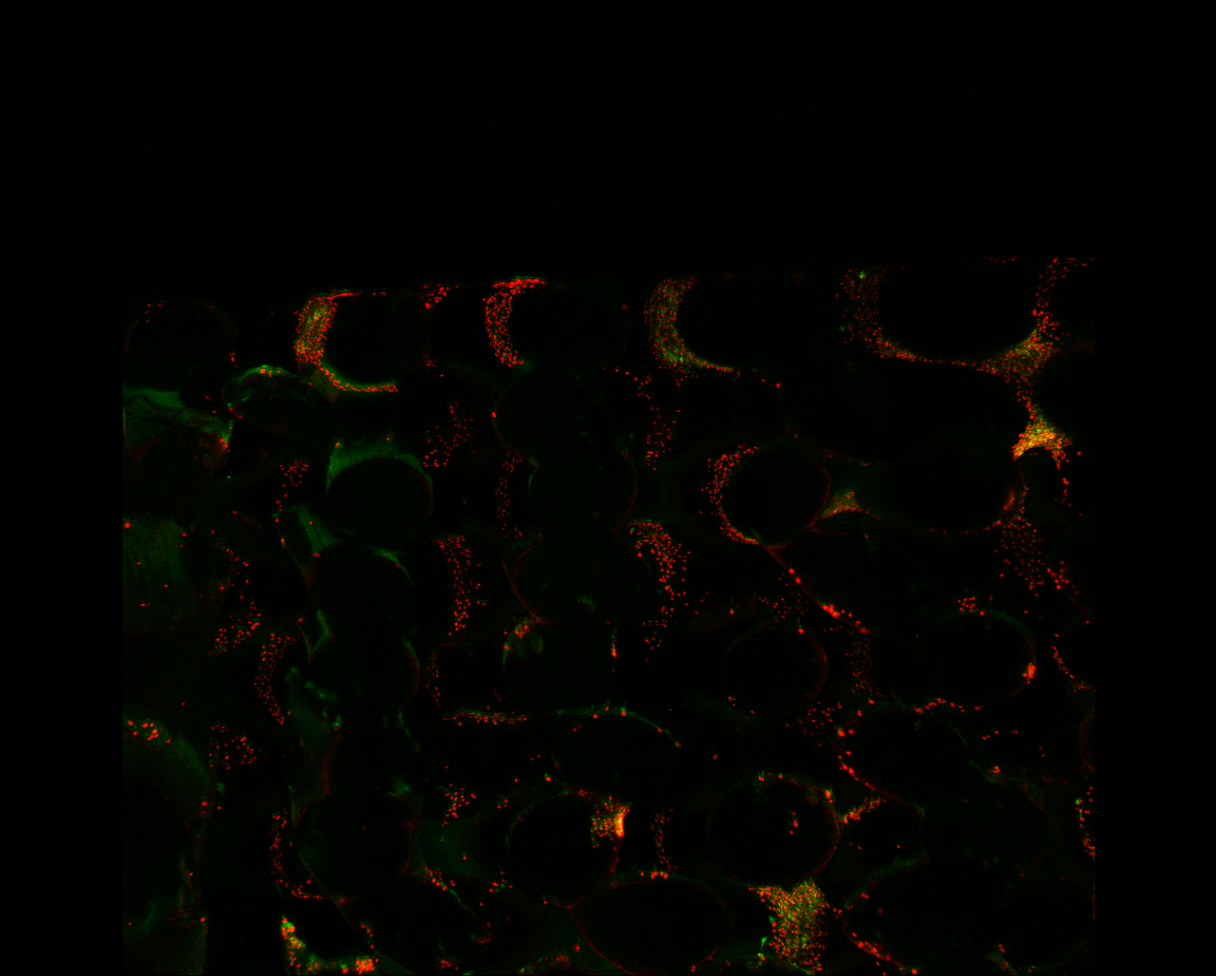

Supplement: Supplementary file 1 [file ijms-24-06040-s001.zip › TGF-H_14Days_Live-Dead.tif]

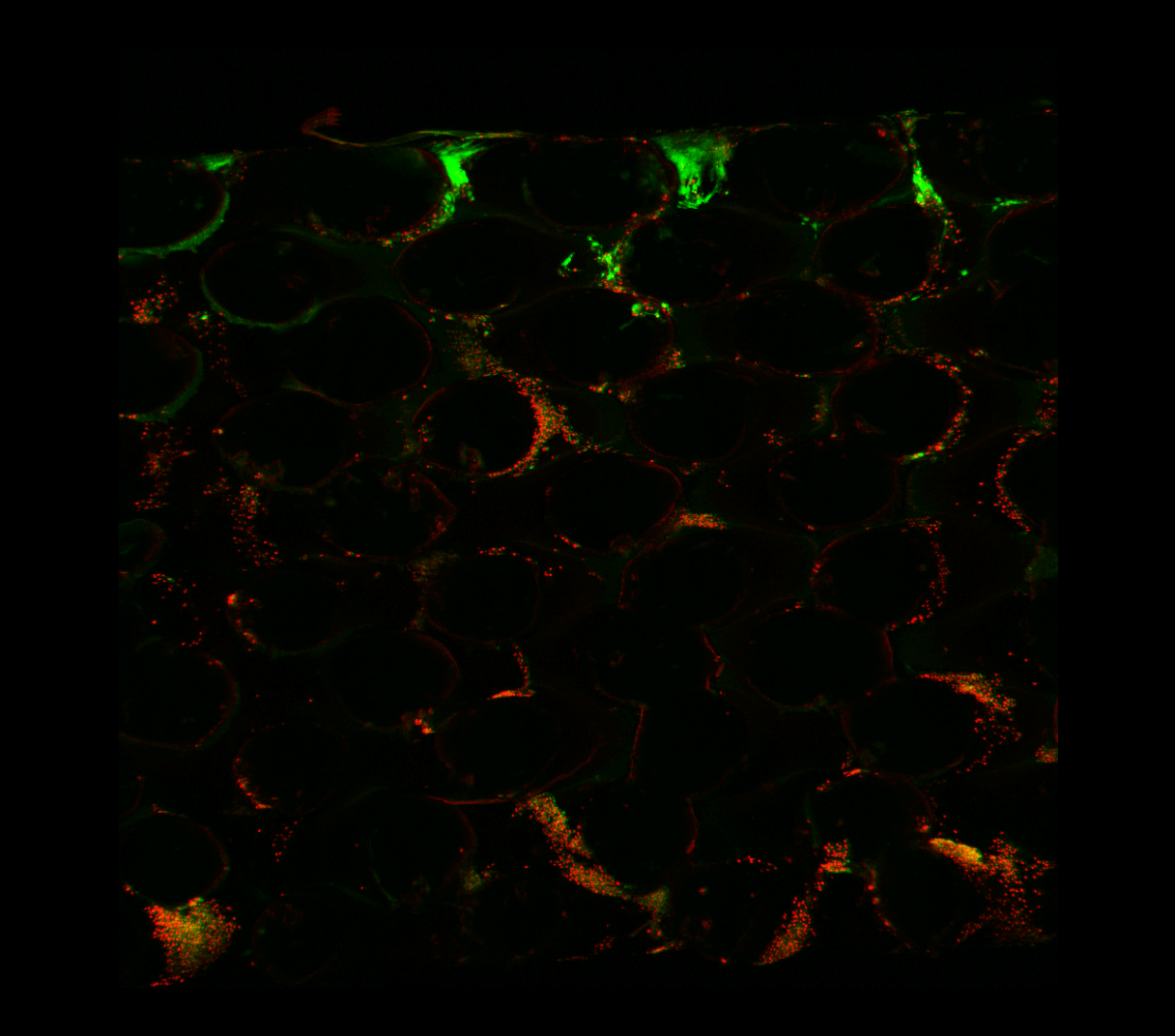

Supplement: Supplementary file 1 [file ijms-24-06040-s001.zip › TGF-H_21Days_Live-Dead.tif]

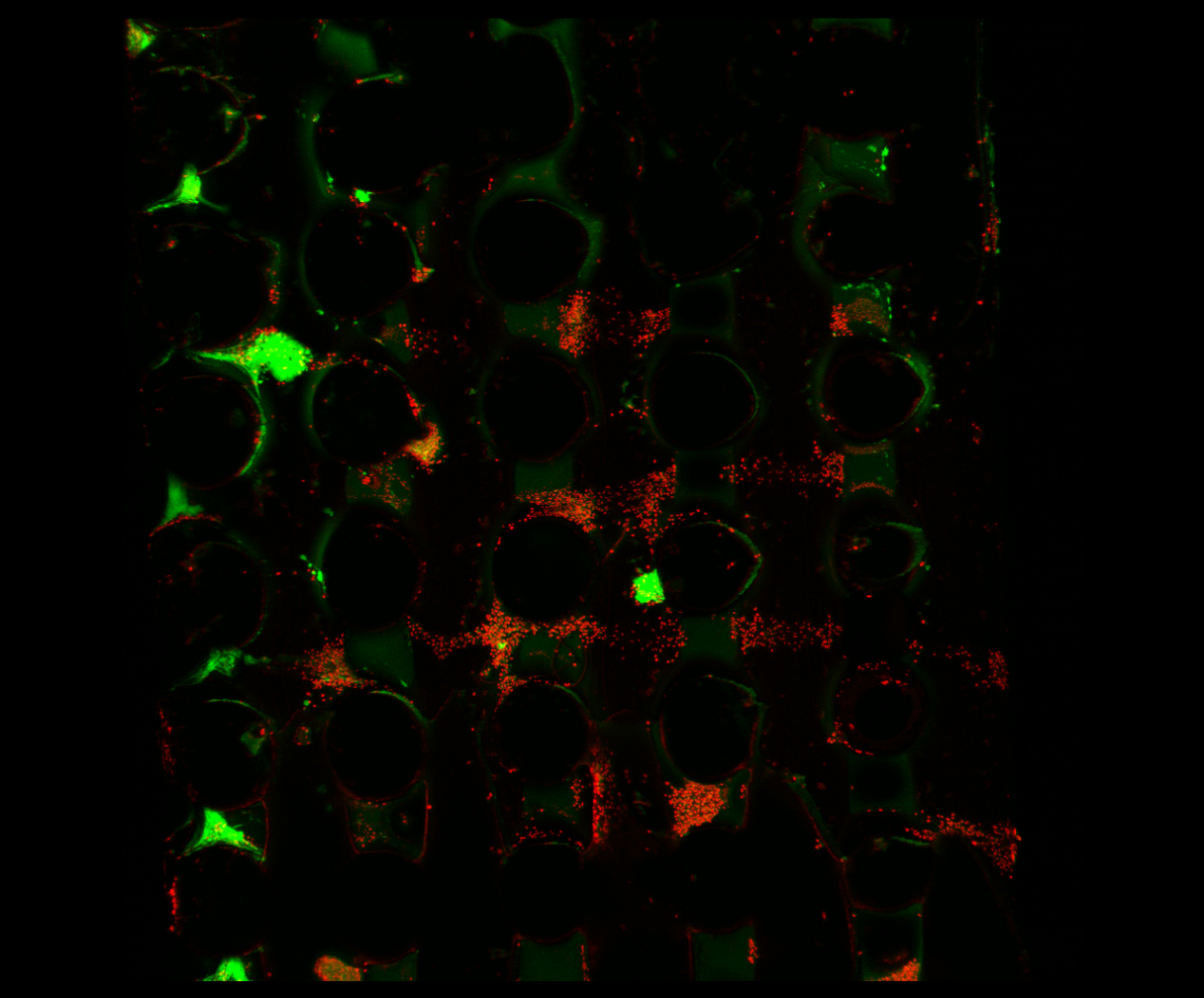

Supplement: Supplementary file 1 [file ijms-24-06040-s001.zip › TGF-H_7Days_ Live-Dead.tif]

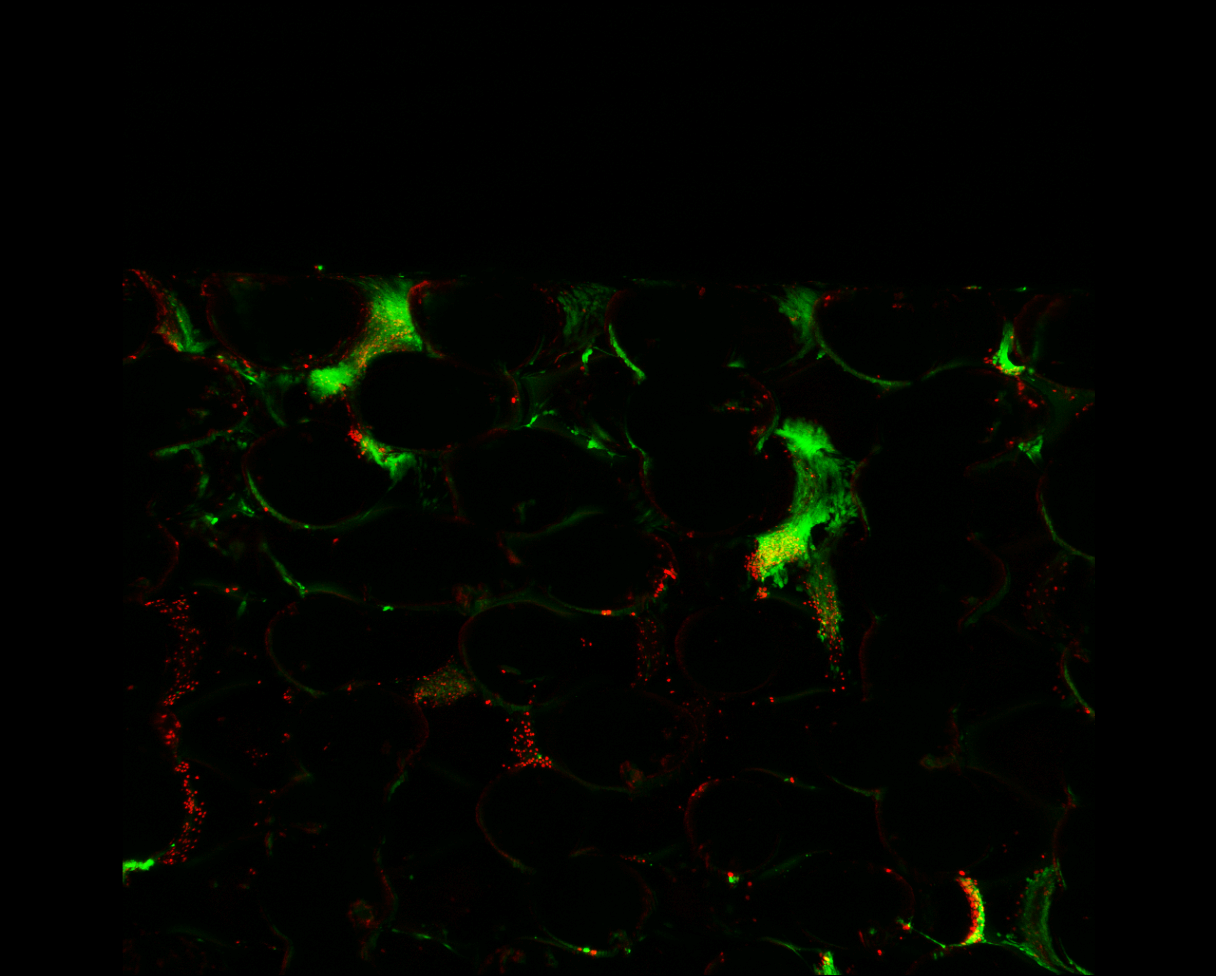

Supplement: Supplementary file 1 [file ijms-24-06040-s001.zip › TGF-N_14Days_Live-Dead.tif]

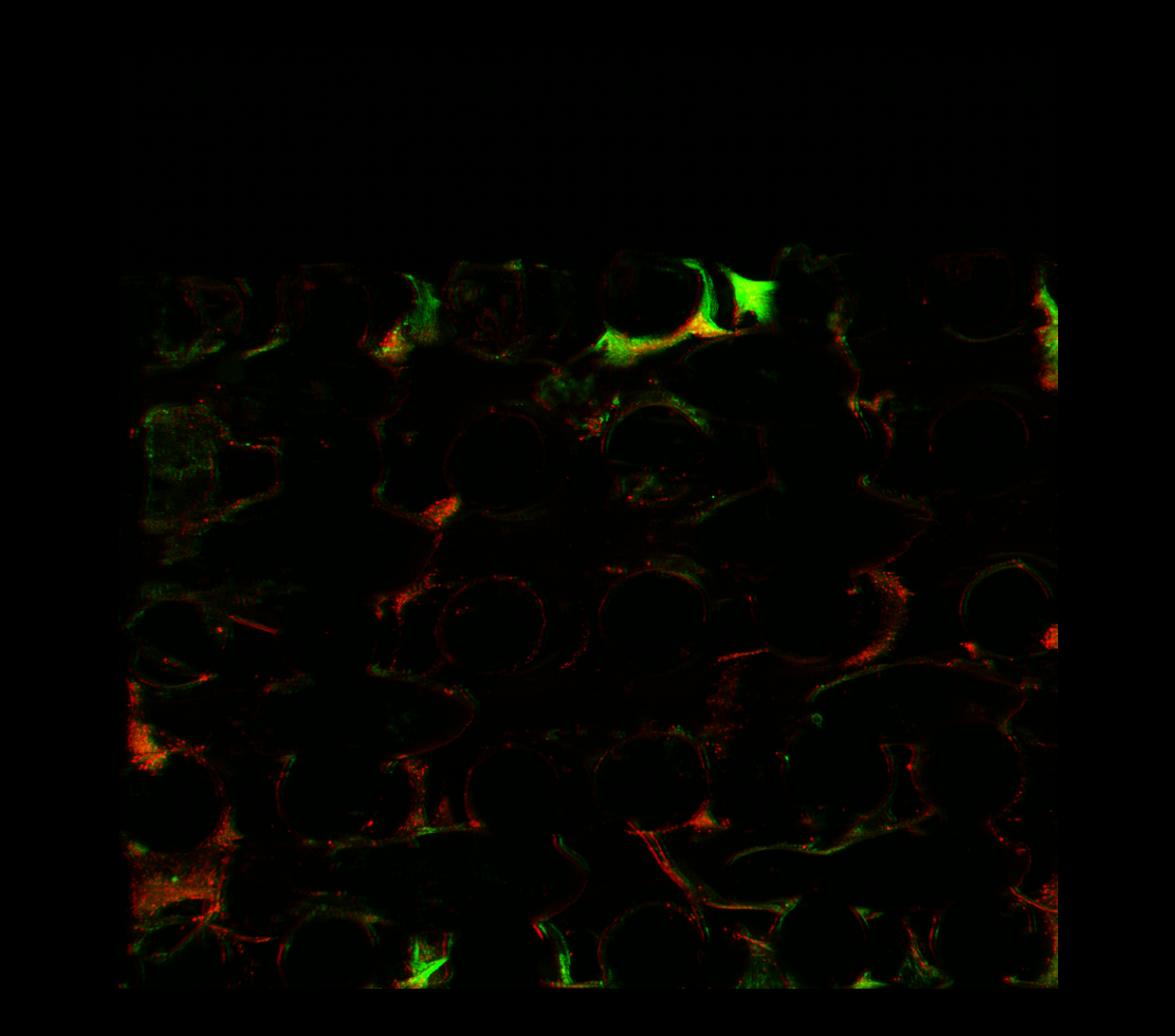

Supplement: Supplementary file 1 [file ijms-24-06040-s001.zip › TGF-N_21Days_Live-Dead.tif]

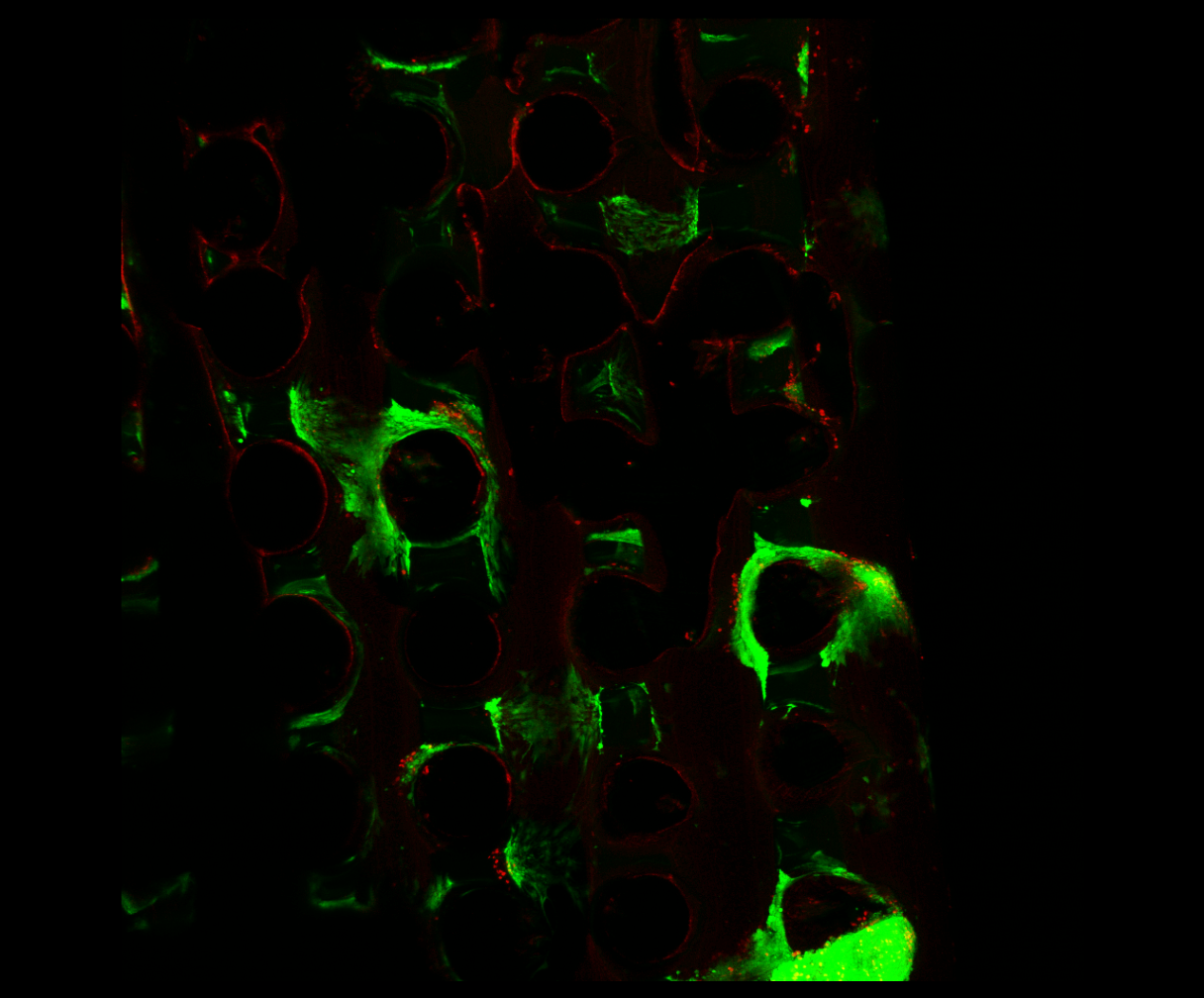

Supplement: Supplementary file 1 [file ijms-24-06040-s001.zip › TGF-N_7Days_Live-Dead.tif]
